# Deciphering the dual roles of PHD finger proteins from oncogenic drivers to tumor suppressors

**DOI:** 10.3389/fcell.2024.1403396

**Published:** 2024-05-15

**Authors:** Tingyu Fan, Lai Jiang, Xuancheng Zhou, Hao Chi, Xi Zeng

**Affiliations:** ^1^ Hunan Province Key Laboratory of Tumor Cellular and Molecular Pathology, Cancer Research Institute, Hengyang Medical School, University of South China, Hengyang, Hunan, China; ^2^ Clinical Medical College, Southwest Medical University, Luzhou, Sichuan, China

**Keywords:** PHD, cancer, signaling pathways, immune responses, oncogenic drivers, tumor suppressors

## Abstract

PHD (plant homeodomain) finger proteins emerge as central epigenetic readers and modulators in cancer biology, orchestrating a broad spectrum of cellular processes pivotal to oncogenesis and tumor suppression. This review delineates the dualistic roles of PHD fingers in cancer, highlighting their involvement in chromatin remodeling, gene expression regulation, and interactions with cellular signaling networks. PHD fingers’ ability to interpret specific histone modifications underscores their influence on gene expression patterns, impacting crucial cancer-related processes such as cell proliferation, DNA repair, and apoptosis. The review delves into the oncogenic potential of certain PHD finger proteins, exemplified by PHF1 and PHF8, which promote tumor progression through epigenetic dysregulation and modulation of signaling pathways like Wnt and TGFβ. Conversely, it discusses the tumor-suppressive functions of PHD finger proteins, such as PHF2 and members of the ING family, which uphold genomic stability and inhibit tumor growth through their interactions with chromatin and transcriptional regulators. Additionally, the review explores the therapeutic potential of targeting PHD finger proteins in cancer treatment, considering their pivotal roles in regulating cancer stem cells and influencing the immune response to cancer therapy. Through a comprehensive synthesis of current insights, this review underscores the complex but promising landscape of PHD finger proteins in cancer biology, advocating for further research to unlock novel therapeutic avenues that leverage their unique cellular roles.

## 1 Introduction

### 1.1 Cellular mechanisms in cancer progression

Cancer presents a significant global health challenge, emerging from DNA alterations that compromise the regulatory frameworks controlling cell proliferation, survival, apoptosis, and DNA repair. This complex disease is propelled by mutations that either suppress tumor activity or activate oncogenic pathways, complicating the understanding of its initiation, progression, and metastasis due to the intricate signaling networks involved (Mantovani et al., 2019). At the heart of these networks lie epigenetic mechanisms, which are processes that alter gene expression without changing the DNA sequence, including DNA methylation, histone modification, and RNA-associated silencing. These epigenetic changes can drastically affect gene expression within signaling networks, leading to the dysregulation of key cellular processes involved in cancer (Mohammad et al., 2019; Ilango et al., 2020; Huang G. et al., 2021). Cancer stem cells (CSCs), a fundamental component of tumors, exhibit self-renewal and differentiation capabilities that drive tumor recurrence, metastasis, and therapy resistance. A promising approach for cancer treatment involves targeting essential signaling pathways and epigenetic regulations to inhibit specific transcription factors and disrupt aberrant CSC metabolism. This strategy holds potential for improving therapeutic outcomes by addressing the unique characteristics of CSCs in cancer progression and treatment resistance (Toh et al., 2017; Yang et al., 2020).

### 1.2 Roles of PHD finger proteins in diseases

Over two decades ago, researchers identified a novel domain in an Arabidopsis protein characterized by its conserved N-terminal cysteine/histidine-rich region, introducing the plant homeodomain (PHD) finger motif to the scientific community (Schindler et al., 1993). The structural integrity of the PHD domain, when paired with a zinc finger motif, encompasses a compact configuration of approximately 60 amino acids. This configuration facilitates chromatin binding and the modulation of gene expression through alterations in chromatin structure (Weissman, 2001). The PHD finger as an epigenetic reader has shown diversity in structural biology, such as the recognition of H3K4 trimethylation, acetyllysine labeling, N-terminal motif binding of the PHD finger, and so on. The folding fine-tuning of histone residues extends the potential of PHD module diversity (Li and Li, 2012). PHD finger proteins are defined by a characteristic “xCDxCDx” motif, with amino acids at binding sites that are enriched, giving rise to diverse molecular subtypes (Boamah et al., 2018). PHD finger proteins do not bind to other efficient structures in a single way. In addition to the typical H3K4me3 histone binding mechanism, non-histone binding, H3 analog binding, and direct contact between lysine residues and DNA have also been found. Different modes of action enhance the connection between PHD domain and chromatin. High throughput screening, proteomics and microarray analysis can be used to calculate the different binding ligands of PHD fingers to assist the study of biological functions (Gaurav and Kutateladze, 2023). These proteins play critical roles in a multitude of cellular functions, ranging from transcription regulation and DNA repair to cell cycle control and developmental growth.

Various types of PHD finger proteins are associated with a spectrum of human conditions, underscoring their fundamental importance in growth and development as well as in disease pathogenesis (Black and Kutateladze, 2023). For instance, PHF2 is essential for maintaining neural development and genomic stability (Pappa et al., 2019). It is also implicated in metabolic disorders, where its regulatory function in gene expression can impact metabolic pathways and insulin sensitivity. Dysregulation of PHF2 activity may induce adipogenesis and metabolic syndrome (Okuno et al., 2013; Jeong et al., 2023). Reprogramming of pluripotent embryonic stem cells is maintained by PHF5A, which stabilizes the transcription program of stem cells and controls the elongation of RNA polymerase II, ensuring the proper realization of stem cell pluripotency and differentiation (Strikoudis et al., 2016). PHF6 and PHF8 are associated with developmental disorders. Mutations in the PHF6 gene are linked to Börjeson-Forssman-Lehmann syndrome, a rare X-linked intellectual disability syndrome characterized by developmental delay, facial dysmorphisms, and other physical anomalies (Hsu et al., 2019).

### 1.3 PHD fingers in cancer development

**FIGURE 1:**
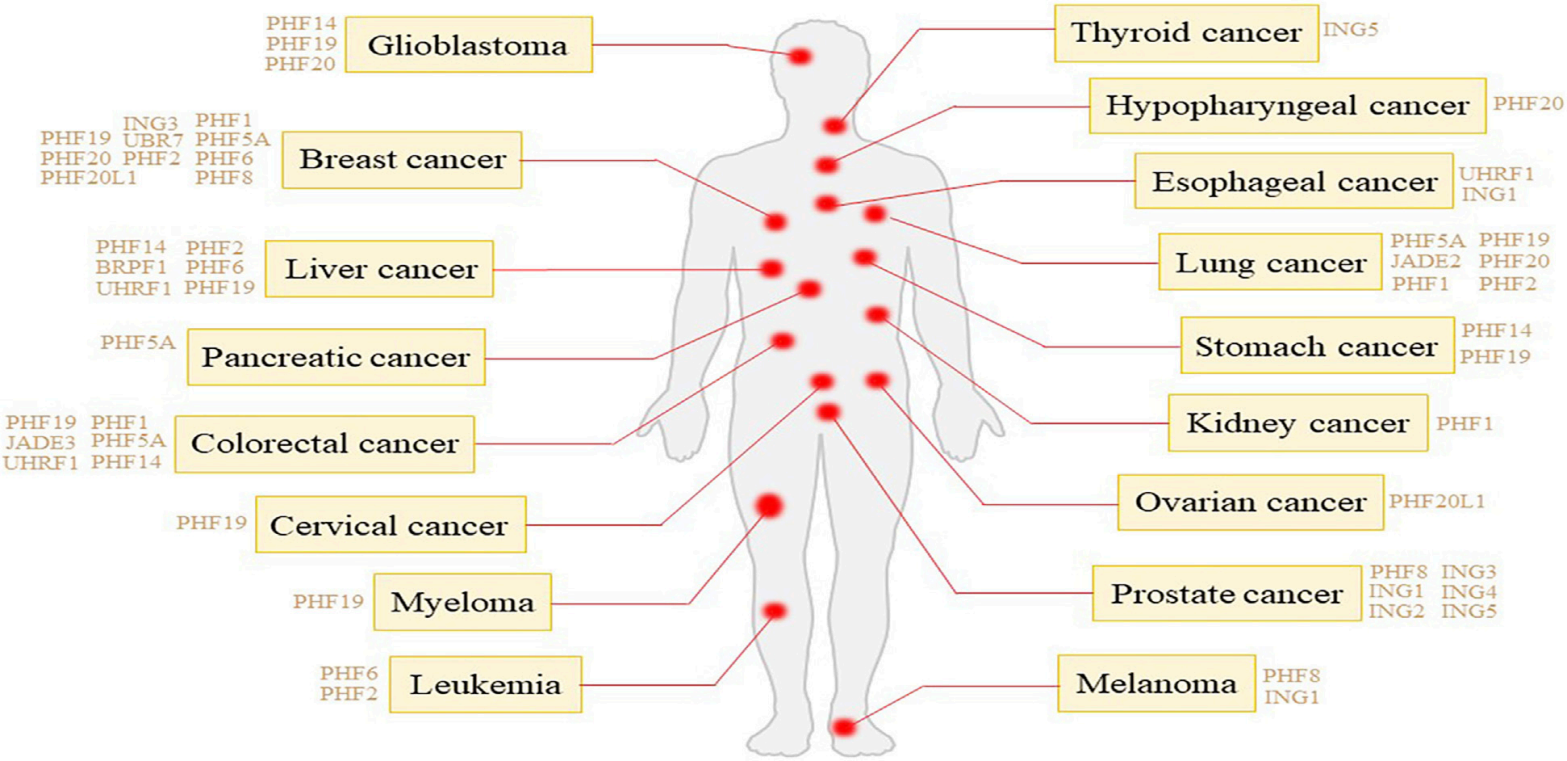
Tumor types associated with different PHD finger proteins.

In the realm of oncology, the significance of PHD fingers extends beyond their mere existence, serving as a critical component in recognizing and interpreting distinct histone modifications such as methylation, acetylation, and phosphorylation. For example, the N-terminal PHD finger of PHF1 recognizes the symmetric demethylation of the third arginine of histone H4 catalyzed by PRMT5-WDR77. The C-terminal PHD is associated with the CUL4B-Ring E3 ligase complex that catalyzes substrate ubiquitination (Liu et al., 2018). PHF6 recruits histone methyltransferase SUV4-20H2, mediating H4K20me3 expression, which is an inhibitory histone modification, and inhibiting rDNA transcription (Huang X. et al., 2021). Interestingly, for histone H2B, PHF6 can both ubiquitinate lysine 120 and acetylate lysine 12, and simultaneously, the identification of the acetylation site helps PHF6 exert its E3 ubiquitin ligase activity (Oh et al., 2020). These modifications are paramount in the regulation of gene expression, achieved by altering the structure and accessibility of chromatin (Jain et al., 2020). As epigenetic markers, the modifications are inheritable through cell divisions, thereby influencing gene expression patterns without altering the underlying DNA sequence. The regulation of epigenetic markers by cancer cells plays a crucial role in their development and progression, affecting the expression of genes involved in cell proliferation, DNA repair, and apoptosis (Chen et al., 2023). PHD finger proteins have been studied in a variety of tumors (Figure 1), such as breast cancer, liver cancer lung cancer and so on. The same PHD finger protein may cause different tumors, and the mechanisms of carcinogenic PHD finger proteins in different tumors may also be different. The intricate interplay of PHD fingers in cancer biology suggests their specific and tissue-dependent roles, acting either as oncogenes or tumor suppressors, depending on the activated signaling pathways in certain cancer types.

ING (Inhibitor of Growth) proteins are vital in cellular functions, bridging histone acetylation activities with chromatin through their conserved region of PHD (Taheri et al., 2022). The PHD mediates interactions with methylated lysine residues in ING proteins, playing a crucial role in gene expression regulation and DNA damage response in cancer biology (Jacquet and Binda, 2021). Recent research elucidates the involvement of PHD finger proteins in the immune response to cancer therapy, shedding light on their impact on treatment effectiveness. For example, interactions between circular RNAs and PHD proteins influence immune responses, impacting the efficacy of immunotherapy (Guan et al., 2023). Although PHD finger proteins are a recent addition to cancer immunotherapy, their capacity to impact cancer development and shape the immune system’s response to treatment warrants further exploration and consideration.

This review aims to consolidate current insights into the role of PHD fingers within cancer-associated mechanisms, emphasizing their significance as oncoproteins, tumor suppressors and therapeutic targets. By elucidating the molecular underpinnings through which PHD fingers modulate tumor progression, this synthesis not only identifies existing knowledge lacunae but also charts a course for future investigational trajectories. The potential unveiling of novel therapeutic avenues through this exploration could significantly pivot cancer treatment paradigms.

## 2 Roles of PHD fingers in oncogenesis

**FIGURE 2:**
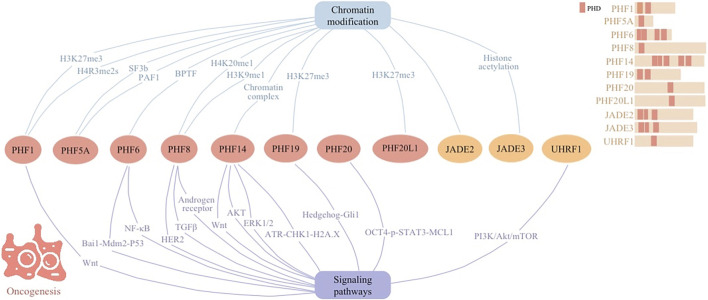
Cellular signaling networks involving plant homeodomain (PHD) finger proteins (PHF1, PHF5A, PHF6, PHF8, PHF14, PHF19, PHF20, and PHF20L1) as well as proteins containing PHD regions (JADE2, JADE3, and UHRF1) in oncogenesis.

PHD fingers intricately regulate chromatin remodeling and gene expression, allowing their significance in both oncogenesis and tumor suppression across various cellular signaling pathways (Figure 2). This positions them as pivotal players in cancer biology. Their involvement spans direct participation in DNA damage repair and cell cycle regulation to indirect roles in shaping the tumor microenvironment and influencing cancer cell metabolism.

### 2.1 Chromatin structure regulation in cancer progression

Dysregulation of PHD finger proteins has been increasingly implicated in the development of cancer. Upregulation of PHF1 in diverse cancers underlines its role in tumorigenesis. PHF1 proceeds epigenetic regulation via its Tudor domain and PHD fingers, recognizing specific histone modifications of arginine 3 on histone H4 (H4R3me2s). Through interactions with protein complex such as PRMT5/WDR77 and CRL4B, PHF1 influences gene networks critical for cell proliferation and migration (Liu et al., 2018). In the development of ossifying fibromyxoid tumor (OFMT), PHF1 modulates transcriptional repression by integrating with the Polycomb Repressive Complex 2 (PRC2), which is responsible for the histone mark lysine 27 on histone H3 (H3K27me3). This impacts chromatin accessibility and enhances oncogenic gene expression, driving cancer progression (Hofvander et al., 2020).

PHF5A, identified as a crucial splicing factor in tumor progression, has been implicated in breast cancer’s aggressive behavior. By stabilizing the SF3b spliceosome and facilitating its interaction with histones, PHF5A alters chromatin structure and gene expression, notably influencing apoptotic pathways (Zheng et al., 2018). This modification, particularly pronounced in breast cancer, underscores PHF5A’s role in undermining apoptosis, highlighting its therapeutic potential against cancer’s advance. Similarly, by interacting with RNA polymerase associated factor PAF1, increased expression of PHF5A correlates with enhanced proliferation and migration in various tumor cell types, including pancreatic tumor (Karmakar et al., 2020), lung cancer (Mao et al., 2019), non-small cell lung cancer (Yang et al., 2022), and colorectal cancer (Wang et al., 2019).

PHF6, initially identified as a tumor suppressor, acts as a prognostic epigenetic regulator in breast cancer. Through its interaction with HIF-1α and HIF-2α, PHF6 enhances HIF-driven transcriptional events to initiate breast tumorigenesis. Additionally, PHF6 recruits Bromodomain PHD finger Transcription Factor (BPTF), a component of the nucleosome remodeling factor complex, to mediate epigenetic remodeling and augment HIF transcriptional activity. High expressions of PHF6 in breast cancer are governed by upstream YAP signals and correlate with a poor prognosis for patients (Gao et al., 2023). Additionally, PHF6 proceeds as an oncogene in hepatocellular carcinoma (HCC), promoting tumor growth and progression. By altering E-cadherin and Vimentin levels upon silencing, PHF6 hints its role in epithelial-to-mesenchymal transition and metastasis (Yu et al., 2019).

PHF8, identified as a histone demethylase, promotes metastasis through its epigenetic regulation abilities. By erasing repressive histone marks of H4 Lysine 20 monomethyl (H4K20me1) and H3 Lysine 9 monomethyl (H3K9me1), PHF8 alters chromatin structure, thereby influencing gene expression (Moubarak et al., 2022). By demethylating and removing repressive histone markers on the promoter region of the FOXA2 gene, PHF8 transcriptionally upregulates FOXA2, which is a key transcription factor involved in neuroendocrine prostate cancer development. This action suggests that PHF8’s function is intertwined with the modification of chromatin structures to regulate gene expression, emphasizing its role in the epigenetic landscape of cancer progression (Liu Q. et al., 2021).

Systematic analysis of data from the Cancer Genome Atlas (TCGA) and the Gene Expression Omnibus (GEO) highlights PHF14’s varied expression across different tumors and its association with patient prognosis. It has found that PHF14 gene amplification is present in many types of tumors, and the genetic mutations dominated by missense mutations also account for a large proportion. Besides, the heat map has showed a positive correlation between PHF14 and three DNA methyltransferases in multiple cancers, which are involved in epigenetic modification of specific bases in DNA sequences. Therefore, PHF14’s involvement in chromatin complex effects underscores its significance in oncogenesis and presents it as a potential target for cancer therapy (Cao et al., 2023). However, the exact histone marker proteins involved with PHF14 for chromatin modification require further investigation.

In multiple myeloma (MM), PHF19 plays a key role in regulating chromatin and consequently the transcriptional landscape by influencing the activity and its recruitment of PRC2. This catalysis of histone mark H3K27me3 affects the expression of crucial genes that govern cell proliferation and differentiation, thereby facilitating cancer progression by altering gene expression patterns and promoting genetic instability within MM cells. Understanding PHF19’s dual role in chromatin remodeling and gene expression provides new avenues for targeted therapies in aggressive MM subsets (Ghamlouch et al., 2021).

As a H3K27me2 reader, PHF20L1 coordinates transcriptional repression by linking PRC2-mediated methylation with NuRD-mediated deacetylation. This function establishes it as a vital contributor to oncogenesis, especially in breast cancer, where it suppresses tumor suppressor genes and promotes cell proliferation and metastasis (Hou et al., 2020). Additionally, PHF20L1 influences the tumor landscape by altering transcriptional dynamics, significantly impacting clinical outcomes and therapy responsiveness in ovarian cancer. The study underscores PHF20L1’s potential as a therapeutic target, given its correlation with diminished patient survival rates (Alberto-Aguilar et al., 2022).

JADE family members JADE2 and JADE3 are key members involved in chromatin remodeling and cell cycle regulation, surface as a hallmark of cancer progression. Findings on non-small cell lung cancer (NSCLC) reveal that JADE2’s heightened expression correlates with improved 5-year survival rates, underscoring its role in tumorigenesis (Murphy et al., 2023). Actual understanding of the specific proteins that co-function with JADE2 to regulate chromatin structure is currently limited. JADE3 contributes to chromatin remodeling through histone acetylation, promoting transcriptional activation of key cancer-related genes. In colon cancer, its upregulation correlates with disease advancement and poorer survival outcomes. By fostering cancer stem cell-like properties through interaction with critical gene promoters, such as LGR5, JADE3 directly influences tumor initiation and growth (Jian et al., 2018).

PHD finger proteins, such as PHF1, PHF5A, PHF6, PHF8, PHF14, PHF19, PHF20L1, JADE2, and JADE3, play critical roles in oncogenesis through modulation of chromatin structure and regulation of cellular signaling networks. By recognizing specific histone modifications, these proteins influence gene networks essential for cell proliferation, migration, and tumorigenesis. For instance, PHF1 engages with PRMT5–WDR77 and CRL4B complexes to promote cancer progression, while PHF5A’s interaction with the SF3b spliceosome and PAF1 impacts apoptotic pathways and cellular behavior across various cancers. Similarly, PHF6, PHF8, and others modulate transcriptional events and chromatin accessibility, driving oncogenic gene expression. The comprehensive involvement of PHD finger proteins in gene regulation through various cellular signaling networks underscore their significance in the epigenetic landscape of cancer progression, offering potential targets for innovative cancer therapies.

### 2.2 Regulation and influence in cancer signaling pathways

The intricate interactions between PHD finger proteins and various signaling pathways elucidate their significant role in tumor growth, metastasis, and the development of resistance to cancer therapies.

PHF1 is involved in multiple gene fusions in the development of OFMT, including EP400–PHF1, MEAF6–PHF1, and PHF1–TFE3, which have profound epigenetic consequences. These fusions significantly affect pathways critical for cell growth and differentiation, including the Wnt signaling pathway. Such alterations in gene expression are largely recapitulated in fibroblast lines expressing these fusions, underscoring the essential role of PHF1 and its associated gene fusions in the oncogenesis of OFMT through modulation of chromatin architecture and transcriptional regulation (Hofvander et al., 2020).

PHF6 mutations, often found alongside JAK3 mutations in T-cell acute lymphoblastic leukemia (T-ALL) patients, play a critical role in cancer progression by modulating signaling pathways. PHF6 deficiency exacerbates JAK3-induced T-ALL by suppressing the Bai1-Mdm2-P53 pathway, independently of the JAK3/STAT5 pathway. This interaction suggests PHF6’s essential function in leukemia development and underscores the potential of combining JAK3 and MDM2 inhibitors as a targeted therapy for T-ALL patients with co-mutation of PHF6 and JAK3 (Yuan et al., 2022). Additionally, PHF6 maintains acute myeloid leukemia (AML) through regulation of the NF-κB signaling pathway. Its depletion inhibits NF-κB signaling by disrupting the PHF6-p50 complex and hindering p50’s nuclear translocation, thereby suppressing BCL2 expression and promoting apoptosis in AML cells. This disruption reduces the proliferation of myeloid leukemia cells and impacts the self-renewal ability of leukemia stem cells (LSCs), suggesting a pro-oncogenic role for PHF6 in myeloid leukemia (Hou et al., 2023).

PHF8 influences prostate cancer progression and resistance mechanisms by integrating into the androgen receptor signaling axis, a key pathway in castration-resistant prostate cancer (CRPC). It is regulated post-transcriptionally by the c-MYC/miR-22 axis, which itself is modulated by androgen receptor signaling. PHF8 co-expression with androgen receptor underscores its role in CRPC, with knockdown studies revealing its significance in cell cycle progression (Maina et al., 2016). Additionally, the role of androgen receptor signaling pathway in breast cancer prognosis and immune infiltration has been previously reported (Huang et al., 2022). PHF8 enhances HER2-positive breast cancer progression through a synergistic interaction with HER2 signaling, regulating key oncogenic pathways. Elevated PHF8 expression, driven by HER2, acts as a coactivator for HER2 transcription, epithelial-to-mesenchymal transition markers, and cytokine production. Specifically, the PHF8-IL-6 axis influences trastuzumab resistance and T-cell infiltration, highlighting its role in tumor immunity and therapy resistance (Liu et al., 2020). Furthermore, PHF8 drives melanoma metastasis by specifically enhancing cell invasion. Through orchestrating a molecular program, PHF8 directly modulates the TGFβ signaling pathway, thereby regulating melanoma invasion and metastasis (Moubarak et al., 2022).

PHF14 emerges as a key regulator in glioblastoma, influencing cancer progression through modulation of the Wnt signaling pathway. Mechanistic insights reveal that PHF14’s regulation of the Wnt pathway alters the expression of key markers in epithelial-mesenchymal transition (EMT) and angiogenesis, highlighting its significance in glioblastoma malignancy and offering a novel therapeutic target avenue (Wu et al., 2019). In colorectal cancer (CRC) progression, PHF14 contributes to cell proliferation and growth, with its knockdown triggering DNA damage and activating the ATR-CHK1-H2A.X pathway, leading to apoptosis (Pan et al., 2022). Furthermore, PHF14 propels tumor progression in gastric cancer by modulating the AKT and ERK1/2 signaling pathways. Attenuation of PHF14 expression results in reduced phosphorylation of AKT and ERK1/2, leading to suppressed colony formation tumorigenesis (Zhao et al., 2020).

PHF19 plays a crucial role in driving CRC advancement by increasing the expression of molecules associated with EMT. Through the modulation of EMT marker expression, PHF19 boosts the migratory and invasive abilities of tumor cells, thereby promoting CRC malignancy (Li et al., 2021). In hepatocellular carcinoma (HCC) progression, PHF19 influences the Hedgehog-Gli1 signaling pathway. By interacting with the E3 ligase of Gli1, PHF19 impedes Gli1 ubiquitination, thereby stabilizing and enhancing Gli1 accumulation The ablation of PHF19 in an HCC mouse model significantly hampers tumorigenesis and prolongs survival, underscoring PHF19’s function as a facilitator of Hedgehog pathway activation (Xiaoyun et al., 2021).

PHF20 promotes apoptosis and enhances cisplatin chemosensitivity in hypopharyngeal squamous cell carcinoma (HSCC) through the OCT4-p-STAT3-MCL1 signaling pathway. Inhibiting PHF20 leads to increased apoptosis, and sensitization to cisplatin mediated via suppression of the OCT4-p-STAT3-MCL1 axis, indicating PHF20’s role in regulating key apoptotic and survival pathways in HSCC (Liu X. et al., 2021).

The ubiquitin-like with plant homeodomain and ring finger domains 1 (UHRF1) regulates cancer signaling pathways in esophageal squamous cell carcinoma (ESCC) by influencing the PI3K/Akt/mTOR pathway, crucial for tumor growth and resistance to therapies. Silencing UHRF1 enhances ESCC cells’ radiosensitivity through apoptosis induction, this process is facilitated by the de-methylation of the tumor suppressor gene PTEN, subsequently inhibiting the PI3K/Akt/mTOR pathway (Hui et al., 2021).

The elucidation of PHD fingers’ regulatory roles across various cancer signaling pathways underscores their key contribution to oncogenesis, tumor progression, and resistance to therapies. These proteins, through their diverse interactions within signaling networks, have emerged as crucial modulators of cancer cell survival, proliferation, metastasis, and therapy response. For instance, PHF6 mutations amplify T-cell acute lymphoblastic leukemia progression by disrupting the Bai1-Mdm2-P53 pathway, independent of the JAK3/STAT5 axis, suggesting a targeted therapeutic strategy involving JAK3 and MDM2 inhibitors. Similarly, PHF8’s involvement in castration-resistant prostate cancer via the AR signaling axis and its role in promoting HER2-positive breast cancer and melanoma metastasis highlight its significance in tumor progression and therapy resistance. Moreover, PHF14 and PHF19 are identified as key players in glioblastoma, colorectal, and hepatocellular cancers, influencing crucial pathways such as Wnt, AKT, ERK1/2, and Hedgehog-Gli1. Additionally, UHRF1’s regulation of the PI3K/Akt/mTOR pathway in esophageal squamous cell carcinoma further illustrates the extensive impact of these proteins on cancer pathophysiology, offering novel insights into potential therapeutic targets.

## 3 Roles of PHD fingers in cancer suppression

**FIGURE 3:**
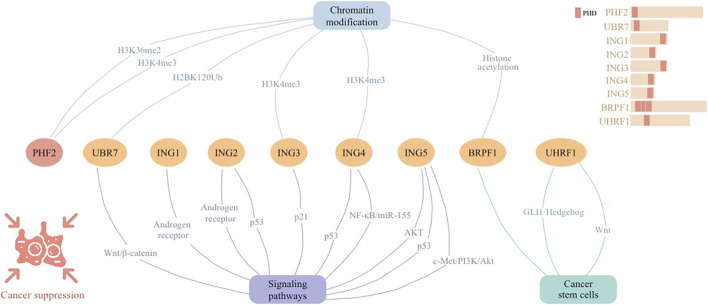
Cellular signaling networks involving plant homeodomain (PHD) finger protein PHF2 as well as proteins containing PHD regions (UBR7, ING1-5, BRPF1, and UHRF1) in cancer suppression.

Tumor-suppressive PHD finger proteins play a crucial role in halting cancer progression through their involvement in chromatin recognition and signaling networks (Figure 3). The ING family proteins, characterized by conserved PHD-type zinc fingers, play pivotal roles in chromatin modification and transcriptional regulation, responding to DNA damage to maintain genomic stability and acting as tumor suppressors in various cancers. By modulating CSCs, PHD fingers pave the way for targeted cancer therapies through epigenetic regulation and signaling pathways. These proteins collectively offer substantial anti-tumorigenic strategies, emphasizing their therapeutic relevance.

### 3.1 PHD finger proteins as tumor suppressor

PHD finger proteins, essential in tumor suppression and epigenetic modulation via intricate cellular signaling networks (Figure 3). Their influence on the epigenetic landscape highlights their significance as potential therapeutic targets, underscoring their foundational importance in cancer biology and treatment paradigms.

PHF2 expression is markedly diminished in ALL patients, correlating with increased leukemia cell proliferation and adverse prognostic markers in B-cell ALL. Functioning as a histone demethylase, PHF2 is implicated in epigenetic transcription regulation. PHF2 reveal its role in counteracting oncogenesis in ALL through the enriched histone mark of H3K4me3. Lower PHF2 levels contribute to leukemia progression. Thus, PHF2 acts as a crucial tumor-inhibiting factor, with its downregulation associated with the aggressive behavior of leukemia cells (Ge et al., 2018). In ovarian cancer (OVCA), the ARID5B-PHF2 complex acts as a tumor-inhibiting factor by promoting histone demethylation at the H3K36me2 site, which enhances the transcription of SORBS2, a gene associated with favorable diagnosis and prognosis. This demethylation activity disrupts EMT process and inhibits tumor progression. The low expression levels of ARID5B, PHF2, and SORBS2 in OVCA tissues and cell lines highlight their potential role in restraining OVCA development, indicating involvement of PHF2 as an anti-oncogene (Deng et al., 2023). Similarly, PHF2 inhibits cancer progression in breast cancer by stimulating the transcription of tumor suppressor genes such as E-cadherin and PHF2 itself, in concert with FOXA2. The interaction between FOXP2 and FOXA2 leads to the activation of PHF2’s transcription, promoting an epithelial phenotype by increasing E-cadherin expression and inhibiting EMT. This regulatory mechanism underlines PHF2’s role in maintaining the epithelial state of breast cancer cells, thereby countering metastatic behaviors and tumor progression (Liu Y. et al., 2021).

The UBR7 PHD finger, characterized by its unique capability to function as an E3 ubiquitin ligase, particularly targets histone H2B at lysine 120 (H2BK120Ub) for monoubiquitination. This enzymatic activity is crucial for suppressing triple-negative breast cancer (TNBC) by regulating histone modifications that impact gene expression critical for tumor suppression and metastasis. The loss of UBR7 is significantly associated with the occurrence and metastatic progression of TNBC, highlighting its role in hampering EMT and tumor generation through epigenetic regulation of key genes involved in cell adhesion and the Wnt/β-catenin signaling pathway (Adhikary et al., 2019).

PHD finger proteins emerge as crucial epigenetic modulators and tumor suppressors across diverse cancers. Their roles in altering the epigenetic landscape underscore their importance in cancer biology. Specifically, PHF2’s reduction correlates with enhanced leukemia proliferation and poor prognosis in B-cell ALL, acting through IKAROS enhancement. In ovarian and breast cancers, PHF2 counteracts oncogenesis by promoting tumor suppressor gene transcription, disrupting EMT. Moreover, UBR7’s unique ubiquitin ligase activity on histone H2BK120 underscores its significance in TNBC suppression through epigenetic regulation. Collectively, these findings highlight PHD finger proteins’ roles in cancer suppression and their potential as therapeutic targets.

### 3.2 ING proteins as multifaced tumor suppressors

The ING family proteins, with their conserved PHD-type zinc fingers, interact with chromatin-modifying complexes, influencing transcriptional and post-translational regulation. ING proteins respond to DNA damage by triggering cell cycle arrest, DNA repair, or apoptosis, thereby maintaining genomic stability and acting as tumor suppressors across various cancers.

ING1 was the pioneering protein in the ING family identified as a tumor suppressor. p33ING1 was shown to impede cell proliferation, whereas its inhibition using antisense RNA resulted in the opposite effect on the growth of breast cancer cells (Garkavtsev et al., 1996). Additionally, ING1 acts as a core component of mSIN3a-HDAC corepressor complexes, contributing to inducing cellular senescence and apoptosis. It is particularly downregulated in castration-resistant prostate cancer cells, highlighting its role in cellular proliferation inhibition and tumor suppression (Melekhova and Baniahmad, 2021).

Similar to its integral role in mSIN3a-HDAC complexes, ING2 modulates cell cycle arrest and apoptosis, especially through the p53 pathway, which underlines its significant function in maintaining cellular integrity and suppressing tumor growth (Melekhova and Baniahmad, 2021). Additionally, ING1 and ING2, through their recruitment by the AR, have been shown to orchestrate the downregulation of the human telomerase reverse transcriptase subunit (hTERT) in prostate cancer cells. Higher androgen levels prompts the engagement of ING1 and ING2 at the hTERT promoter, leading to its repression. Such a mechanism underscores the complex interplay between hormonal signaling and epigenetic modifiers in cancer progression, where ING1 and ING2 emerge as critical mediators of AR’s transcriptional repressive functions on key oncogenic drivers (Bartsch et al., 2021).

This function of ING3 is notable in breast cancer, where nuclear ING3 expression is considerably reduced compared to normal tissues. Its lower nuclear presence is significantly associated with adverse clinicopathological characteristics such as high histological grade and lymph node metastasis, highlighting its role in cancer suppression. High nuclear ING3 levels are linked with improved prognosis, suggesting its potential as a prognostic indicator in breast cancer management (Wu et al., 2020). Additionally, ING3 is linked with poor disease prognosis in specific prostate cancer subgroups. It plays a key role in cellular senescence and apoptosis, particularly through p21 upregulation (Melekhova and Baniahmad, 2021). Binding of ING3 with histone mark H3K4me3 prevents EMT in prostate cancer cells (Melekhova et al., 2021).

Known for its tumor-suppressing capabilities, ING4’s expression is diminished in a significant portion of primary malignancies in prostate cancer. It modulates key processes such as angiogenesis, cellular senescence, and the NF-κB/miR-155 pathway, thereby influencing tumor growth and invasion (Melekhova and Baniahmad, 2021). Similarly, ING4 suppresses tumorigenesis by promoting pathologic cell cycle arrest, apoptosis, autophagy, contact inhibition, and hypoxic adaptation, while impeding tumor angiogenesis, invasion, and metastasis. These effects are mediated through chromatin acetylation by binding to histone H3 trimethylated at lysine 4 (H3K4me3), and through transcriptional regulation of key factors like P53 and NF-κB in various human cancers (Du et al., 2019).

In prostate cancer, ING5’s involvement in apoptosis, particularly in androgen-dependent and -independent prostate cancer cell lines, underscores its crucial role in tumor suppression (Melekhova and Baniahmad, 2021). Besides, ING5 significantly downregulates prostate cancer and curbs tumor growth by inhibiting cell proliferation, clonogenicity, migration, and invasion, while promoting apoptosis. This tumor-restraining effect is mediated through the attenuation of AKT signaling and activation of the p53 pathway (Barlak et al., 2020). Furthermore, ING5 markedly reduces hepatocyte growth factor-induced proliferation, invasion, and epithelial-mesenchymal transition in thyroid cancer cells, and diminishes tumor growth and metastasis *in vivo*. This effect is mediated through the c-Met/PI3K/Akt signaling pathway, highlighting ING5’s role in inhibiting thyroid cancer progression (Gao and Han, 2018).

PHD fingers, including the ING family, are integral to histone remodeling and contribute to various cancer signaling pathways, orchestrating responses to DNA damage or apoptosis in malignant cells. ING1 and ING2, key components in mSIN3a-HDAC complexes, actively repress cell proliferation and tumor growth, also modulating hormonal-epigenetic interplay via androgen receptor signaling. ING3’s diminished nuclear expression in breast cancer correlates with aggressive disease via p21 signaling, while ING4’s involvement in multiple regulatory pathways, including the NF-κB/miR-155 axis, mitigates tumor progression. ING5’s downregulation attenuates oncogenic signaling, exemplified by its counteractive effects on the c-Met/PI3K/Akt pathway in thyroid cancer. Collectively, these proteins are essential for the negative regulation of cancer cell proliferation and the advancement of apoptosis, positioning the ING family as substantial contributors to tumor suppression mechanisms.

### 3.3 Regulating cancer stem cells

Exploration of PHD finger proteins has unveiled their significance in the modulation of CSCs, heralding a new era in the development of targeted cancer therapies. These proteins, integral to the machinery of epigenetic regulation and signaling pathway interactions, have been pinpointed as crucial agents in influencing the behavior of CSCs, thus opening novel avenues for therapeutic intervention.

The investigation into liver cancer CSCs has revealed the therapeutic promise of targeting PHD finger proteins. PHD finger-containing protein BRPF1 contributes to histone acetylation. Significant elevation of BRPF1 in CD133+ liver CSCs, when inhibited by compounds like GSK5959, leads to a notable decline in the sphere-forming ability of liver cancer cells. This reduction is a critical marker of the diminished proliferative and survival capabilities of CSCs, suggesting a viable strategy for combating liver cancer, a condition notorious for its aggressive progression and limited treatment avenues (Cheng et al., 2021).

Moreover, targeting PHD finger-containing protein UHRF1 markedly slows the advancement of hepatocellular carcinoma. UHRF1 contributes to inhibition of GLI1/Hedgehog and Wnt signaling, resulting in decreased tumor growth and a shift in the expression profile of CSC-specific genes. This highlights UHRF1’s potential as a therapeutic target, offering new hope for enhanced treatment outcomes in hepatocellular carcinoma cases (Wang et al., 2023).

PHD finger proteins, through their roles in histone modification and signaling pathways, present novel therapeutic targets against CSCs in liver cancer. The inhibition of BRPF1 and UHRF1, both containing PHD, overexpressed in liver CSCs, reduces CSC proliferation and survival, curbing tumor growth. These findings underscore the potential of PHD finger proteins in altering CSC characteristics and improving hepatocellular carcinoma treatment strategies.

### 3.4 Interaction of PHD fingers with immune regulation

PHD finger proteins are emerging as critical epigenetic modulators in cancer immunology. Their elevated expression in various cancers aligns with increased immune response, underscoring their capacity to modulate tumor immunity, shaping reactions to cancer treatment and providing promising targets for enhancing immunotherapeutic efficacy.

IL6, an inflammatory cytokine released by immune cells, links CD8^+^ Teff trafficking across tumor vessels to antigen-specific tumor apoptotic targets (Fisher et al., 2011). In HER2-positive breast cancer, IL6 has a central position in the PHF8 differentially regulatory protein network. In addition, trastuzumab induced apoptosis increased when PHF8 was knocked down, and this state was reversed with the addition of IL6 (Liu et al., 2020). Exploring the interaction between PHD finger proteins and inflammatory factors is helpful to discover the window of tumor therapy mediated by immune T cells.

Cancer-associated fibroblasts (CAFs) coordinate the function of immune cell infiltration in the tumor microenvironment (Sahai et al., 2020). Algorithmic analysis implied at the relationship between PHF14 and CAFs, enriching the hypothesis that PHF14 affects immune regulation. Further studies showed that PHF14’s expression is positively correlated with tumor immune checkpoint gene expression levels, indicating its significant role in modulating tumor immunity. By regulating the expression of immune checkpoint genes, PHF14 potentially influences the immune system’s ability to recognize and combat tumor cells. This interaction suggests a mechanism through which PHF14 may contribute to tumor immune escape, highlighting its importance in the context of immunology and cancer progression (Cao et al., 2023). Understanding PHF14’s influence on immune checkpoint pathways could enhance the efficacy of immune checkpoint inhibitors, offering new avenues for cancer immunotherapy.

PHF19, a key component of PRC2, is identified as an epigenetic regulator implicated in various cancers, including HCC. The upregulation of PHF19 in cancers correlates with increased immune cell infiltration, particularly of myeloid-derived suppressor cells and Th2 subsets of CD4^+^ T cells, which may contribute to the immune evasion mechanisms of tumors. The expression of PHF19 is also significantly associated with tumor mutation burden and microsatellite instability, suggesting a potential role in the modulation of immune response to cancer (Zhu et al., 2021). This relationship underscores the potential of targeting PHD finger proteins to overcome immune suppression and enhance the efficacy of cancer therapies.

PHD finger proteins, particularly PHF14 and PHF19, exhibit a profound capacity to regulate immune cell infiltration and checkpoint gene expression within tumors, thereby influencing the immune landscape of various cancers, including hepatocellular carcinoma. Their upregulation, correlating with tumor mutation burden and microsatellite instability, implies a role in immune modulation and cancer progression. The association between PHF14 and immune checkpoints suggests a potential to modulate the tumor’s visibility to the immune system. Given these proteins’ influence on cancer immunity, they merit increased attention as targets to enhance immunotherapy responses, opening new research directions in cancer immunology.

## 4 Synopsis of the dual roles of PHD finger

PHD finger proteins are integral components of the cellular machinery, modulating chromatin structure to regulate gene expression. In oncogenesis, PHD finger proteins like PHF1, PHF5A, and PHF8 contribute to cancer progression by dysregulating chromatin (Liu et al., 2018; Karmakar et al., 2020; Moubarak et al., 2022). For example, PHF1 is implicated in tumorigenesis through its role in epigenetic regulation, recognizing specific histone modifications and influencing gene networks essential for cell proliferation and migration (Liu et al., 2018). Similarly, PHF5A, by modulating splicing and chromatin structure, affects gene expression that undermines apoptosis, promoting cancer progression (Zheng et al., 2018).

Conversely, the tumor-suppressive role of PHD finger proteins is exemplified by PHF2 and the ING family, which maintain genomic stability and suppress tumor growth through chromatin modification and transcriptional regulation. PHF2 acts as a histone demethylase, reducing leukemia cell proliferation by influencing histone marks and tumor suppressor gene transcription. The ING family, with conserved PHD-type zinc fingers, participates in histone remodeling and chromatin-modifying complexes, acting as tumor suppressors (Jeong et al., 2023).

PHD finger proteins operate through diverse signaling pathways to dictate cancer dynamics. For oncogenesis, proteins such as PHF1, PHF6, PHF8, PHF14, PHF19 and PHF20 contribute to cancer progression by modulating key signaling pathways including Wnt, Bai1-Mdm2-P53, NF-κB, HER2, TGFβ, androgen receptor and AKT, influence gene networks crucial for cell proliferation (Maina et al., 2016; Hofvander et al., 2020; Liu et al., 2020; Zhao et al., 2020; Moubarak et al., 2022; Yuan et al., 2022; Hou et al., 2023). The Wnt signaling pathway is implicated in the progression of OFMT and glioblastoma through the involvement of both PHF1 and PHF14 (Wu et al., 2019). Similarly, PHD-containing protein UHRF1 regulates esophageal squamous cell carcinoma via the PI3K/Akt/mTOR pathway, facilitated by the demethylation of tumor suppressor genes (Hui et al., 2021).

On the other hand, certain PHD fingers act as tumor suppressors. PHD-containing protein UBR7, for instance, is implicated in TNBC through its Wnt/β-catenin (Adhikary et al., 2019). The ING family proteins, characterized by their PHD fingers, further exemplify this tumor-suppressive role. ING1 and ING2, for example, modulate cell cycle arrest and apoptosis through androgen receptor signalling, contributing to tumor suppression (Bartsch et al., 2021). The p53 signaling pathway is implicated in suppressing prostate cancer and various other cancer types by engaging with ING2, ING4, and ING5.

As for drug therapy research, at present, the targeted drugs of the PHD domain are still unclear, but small molecule inhibitors and peptides that regulate the histone binding activity of the PHD finger have been designed (Table 1). The PHD domain’s primary function is to recognize histone modifications, affecting post-transcriptional translation. Besides, it can also fulfil functions in combination with DNA sequences. Fragment-based NMR screening to identify benzimidazole, which docked in H3K4me-specific pockets and replaced the H3K4me peptide on PHD fingers (Miller et al., 2014). Elise K. Wagner et al. used the newly developed HaloTag technique to identify, amiodarone, tegaserod and disulfiram as inhibitors that disrupt the binding of JARID1A PHD3 and H3K4me3 (Wagner et al., 2012). The combination of PHD fingers with histone H3 methylated lysine 4 can be interrupted by Calixarenes (Ali et al., 2015). A compound, 4-benzylpiperidin-1-carboximidamide, was found by small molecule fragment screening to reduce the affinity of PHD to the H3K9me3 peptides (Houliston et al., 2017). These small molecules allosteric targeting can provide a template for the study of PHD finger protein-related tumor inhibitors.

**TABLE 1:** PHD domain-related drugs/chemicals and their mechanisms of action.

Drugs/chemicals	Mechanisms of action	References
Benzothiazole derivatives	Competitive binding to the K4me pocket	Miller et al. (2014)
Amiodarone	Electrostatic contacts between inhibitor amine groups and the carboxyl group of the aspartate side chain	Wagner et al. (2012)
Tegaserod		
Disulfiram	Ejection of structural zinc	
Calixarenes	Preferentially disrupting PHD-H3K4me3 complexes	Ali et al. (2015)
4-Benzylpiperidine-1-Carboximidamide (BPC)	Reducing PHD affinity with H3K9me3 peptides	Houliston et al. (2017)

Moreover, the correlation between PHF14 expression and immune checkpoint gene levels suggests a potential for PHD finger proteins to influence the tumor immune environment, offering insights into enhancing the efficacy of immunotherapeutic agents (Cao et al., 2023). By navigating the intricate landscape of chromatin structure and cellular signaling networks, PHD fingers offer a promising target for innovative cancer therapies. Harnessing the potential of PHD finger proteins to modulate the epigenetic and signaling pathways in cancer not only deepens our understanding of tumor biology but also paves the way for revolutionizing cancer treatment strategies.

## 5 Conclusion

The PHD finger proteins have carved a niche in the realm of cancer biology, revolutionizing treatment approaches through their nuanced regulation of cellular signaling networks. By modulating chromatin structure and gene expression, PHD fingers act at the crossroads of oncogenesis and tumor suppression, offering new therapeutic avenues. These proteins not only participate directly in DNA repair and cell cycle regulation but also exert indirect effects on the tumor microenvironment and cancer cell metabolism, underscoring their integral role in cancer progression.

In oncogenesis, PHD fingers are implicated in enhancing tumor growth and metastasis through aberrant epigenetic regulation. Conversely, as tumor suppressors, they maintain genomic stability, influencing key processes like apoptosis and cell proliferation. Their versatility is further highlighted in their interaction with CSCs, pointing towards strategies that target CSC-related pathways for cancer treatment, potentially curbing tumor recurrence and metastasis.

The dual functionality of PHD finger proteins in cancer underscores the complexity of their roles in cellular signaling pathways. On one hand, mutations in PHD finger proteins like PHF6 exacerbate leukemia progression, while on the other hand, proteins such as UHRF1 regulate essential pathways in ESCC, affecting tumor growth and therapy resistance. This intricate interplay positions PHD fingers as critical modulators of cancer cell survival, proliferation, and metastasis, as well as therapy response.

Looking ahead, PHD finger proteins hold the promise of enhancing immunotherapeutic efficacy. Their impact on immune cell infiltration and the expression of immune checkpoint genes provides a novel perspective on modulating tumor immunity. Targeting PHD finger proteins to overcome immune suppression could significantly improve cancer treatment outcomes, emphasizing their potential as therapeutic targets. In addition, signaling pathway inhibitors and epigenetic drugs that could be used in clinical treatment also have well research prospects.

In conclusion, PHD finger proteins represent a fascinating and complex element in cancer biology. Their ability to navigate and modulate intricate signaling networks offers groundbreaking potential in cancer treatment. The exploration of PHD fingers in oncology opens up a spectrum of therapeutic possibilities, promising to shift paradigms in cancer treatment. Further research into these proteins will undoubtedly yield profound insights, paving the way for novel therapies that leverage their unique roles in the cellular regulation of cancer.

## References

[B1] Adhikary S. Chakravarti D. Terranova C. Sengupta I. Maitituoheti M. Dasgupta A. (2019). Atypical plant homeodomain of UBR7 functions as an H2BK120Ub ligase and breast tumor suppressor. Nat. Commun. 10 (1), 1398. 10.1038/s41467-019-08986-5 30923315 PMC6438984

[B2] Alberto-Aguilar D. R. Hernández-Ramírez V. I. Osorio-Trujillo J. C. Gallardo-Rincón D. Toledo-Leyva A. Talamás-Rohana P. (2022). PHD finger protein 20-like protein 1 (PHF20L1) in ovarian cancer: from its overexpression in tissue to its upregulation by the ascites microenvironment. Cancer Cell Int. 22 (1), 6. 10.1186/s12935-021-02425-6 34991589 PMC8740351

[B3] Ali M. Daze K. D. Strongin D. E. Rothbart S. B. Rincon-Arano H. Allen H. F. (2015). Molecular insights into inhibition of the methylated histone-plant homeodomain complexes by Calixarenes. J. Biol. Chem. 290 (38), 22919–22930. 10.1074/jbc.M115.669333 26229108 PMC4645621

[B4] Barlak N. Capik O. Sanli F. Kilic A. Aytatli A. Yazici A. (2020). ING5 inhibits cancer aggressiveness by inhibiting Akt and activating p53 in prostate cancer. Cell Biol. Int. 44 (1), 242–252. 10.1002/cbin.11227 31475765

[B5] Bartsch S. Mirzakhani K. Neubert L. Stenzel A. Ehsani M. Esmaeili M. (2021). Antithetic hTERT regulation by androgens in prostate cancer cells: hTERT inhibition is mediated by the ING1 and ING2 tumor suppressors. Cancers (Basel) 13 (16), 4025. 10.3390/cancers13164025 34439179 PMC8391603

[B6] Black J. C. Kutateladze T. G. (2023). Atypical histone targets of PHD fingers. J. Biol. Chem. 299 (4), 104601. 10.1016/j.jbc.2023.104601 36907441 PMC10124903

[B7] Boamah D. Lin T. Poppinga F. A. Basu S. Rahman S. Essel F. (2018). Characteristics of a PHD finger subtype. Biochemistry 57 (5), 525–539. 10.1021/acs.biochem.7b00705 29253329 PMC6048597

[B8] Cao Z. Zhan H. Wu W. Kuang Z. Mo F. Liu X. (2023). A comprehensive pan-cancer analysis unveiling the oncogenic effect of plant homeodomain finger protein 14 (PHF14) in human tumors. Front. Genet. 14, 1073138. 10.3389/fgene.2023.1073138 37007943 PMC10061232

[B9] Chen X. Xu H. Shu X. Song C. X. (2023). Mapping epigenetic modifications by sequencing technologies. Cell Death Differ. 2023, 01213. 10.1038/s41418-023-01213-1 PMC11742697 37658169

[B10] Cheng C. L. Tsang F. H. Wei L. Chen M. Chin D. W. Shen J. (2021). Bromodomain-containing protein BRPF1 is a therapeutic target for liver cancer. Commun. Biol. 4 (1), 888. 10.1038/s42003-021-02405-6 34285329 PMC8292510

[B11] Deng Y. Dong Y. Wu L. Zhang Q. Yang L. (2023). ARID5B promoted the histone demethylation of SORBS2 and hampered the metastasis of ovarian cancer. Pathol. Res. Pract. 252, 154911. 10.1016/j.prp.2023.154911 37948999

[B12] Du Y. Cheng Y. Su G. (2019). The essential role of tumor suppressor gene ING4 in various human cancers and non-neoplastic disorders. Biosci. Rep. 39 (1). 10.1042/bsr20180773 PMC6356015 30643005

[B13] Fisher D. T. Chen Q. Skitzki J. J. Muhitch J. B. Zhou L. Appenheimer M. M. (2011). IL-6 trans-signaling licenses mouse and human tumor microvascular gateways for trafficking of cytotoxic T cells. J. Clin. Invest. 121 (10), 3846–3859. 10.1172/jci44952 21926464 PMC3195455

[B14] Gao S. Zhang W. Ma J. Ni X. (2023). PHF6 recruits BPTF to promote HIF-dependent pathway and progression in YAP-high breast cancer. J. Transl. Med. 21 (1), 220. 10.1186/s12967-023-04031-8 36967443 PMC10040131

[B15] Gao W. Han J. (2018). Overexpression of ING5 inhibits HGF-induced proliferation, invasion and EMT in thyroid cancer cells via regulation of the c-Met/PI3K/Akt signaling pathway. Biomed. Pharmacother. 98, 265–270. 10.1016/j.biopha.2017.12.045 29272787

[B16] Garkavtsev I. Kazarov A. Gudkov A. Riabowol K. (1996). Suppression of the novel growth inhibitor p33ING1 promotes neoplastic transformation. Nat. Genet. 14 (4), 415–420. 10.1038/ng1296-415 8944021

[B17] Gaurav N. Kutateladze T. G. (2023). Non-histone binding functions of PHD fingers. Trends Biochem. Sci. 48 (7), 610–617. 10.1016/j.tibs.2023.03.005 37061424 PMC10330121

[B18] Ge Z. Gu Y. Han Q. Sloane J. Ge Q. Gao G. (2018). Plant homeodomain finger protein 2 as a novel IKAROS target in acute lymphoblastic leukemia. Epigenomics 10 (1), 59–69. 10.2217/epi-2017-0092 28994305 PMC5992565

[B19] Ghamlouch H. Boyle E. M. Blaney P. Wang Y. Choi J. Williams L. (2021). Insights into high-risk multiple myeloma from an analysis of the role of PHF19 in cancer. J. Exp. Clin. Cancer Res. 40 (1), 380. 10.1186/s13046-021-02185-1 34857028 PMC8638425

[B20] Guan L. Hao Q. Shi F. Gao B. Wang M. Zhou X. (2023). Regulation of the tumor immune microenvironment by cancer-derived circular RNAs. Cell Death Dis. 14 (2), 132. 10.1038/s41419-023-05647-w 36797245 PMC9935907

[B21] Hofvander J. Jo V. Y. Fletcher C. D. M. Puls F. Flucke U. Nilsson J. (2020). PHF1 fusions cause distinct gene expression and chromatin accessibility profiles in ossifying fibromyxoid tumors and mesenchymal cells. Mod. Pathol. 33 (7), 1331–1340. 10.1038/s41379-020-0457-8 31932680

[B22] Hou S. Wang X. Guo T. Lan Y. Yuan S. Yang S. (2023). PHF6 maintains acute myeloid leukemia via regulating NF-κB signaling pathway. Leukemia 37 (8), 1626–1637. 10.1038/s41375-023-01953-6 37393343 PMC10400421

[B23] Hou Y. Liu W. Yi X. Yang Y. Su D. Huang W. (2020). PHF20L1 as a H3K27me2 reader coordinates with transcriptional repressors to promote breast tumorigenesis. Sci. Adv. 6 (16), eaaz0356. 10.1126/sciadv.aaz0356 32494608 PMC7159910

[B24] Houliston R. S. Lemak A. Iqbal A. Ivanochko D. Duan S. Kaustov L. (2017). Conformational dynamics of the TTD-PHD histone reader module of the UHRF1 epigenetic regulator reveals multiple histone-binding states, allosteric regulation, and druggability. J. Biol. Chem. 292 (51), 20947–20959. 10.1074/jbc.M117.799700 29074623 PMC5743070

[B25] Hsu Y. C. Chen T. C. Lin C. C. Yuan C. T. Hsu C. L. Hou H. A. (2019). Phf6-null hematopoietic stem cells have enhanced self-renewal capacity and oncogenic potentials. Blood Adv. 3 (15), 2355–2367. 10.1182/bloodadvances.2019000391 31395598 PMC6693005

[B26] Huang G. Cao H. Liu G. Chen J. (2022). Role of androgen receptor signaling pathway-related lncRNAs in the prognosis and immune infiltration of breast cancer. Sci. Rep. 12 (1), 20631. 10.1038/s41598-022-25231-0 36450882 PMC9712677

[B27] Huang G. Chen J. Zhou J. Xiao S. Zeng W. Xia J. (2021a). Epigenetic modification and BRAF gene mutation in thyroid carcinoma. Cancer Cell Int. 21 (1), 687. 10.1186/s12935-021-02405-w 34923978 PMC8684614

[B28] Huang X. Zhang X. Zong L. Gao Q. Zhang C. Wei R. (2021b). Gene body methylation safeguards ribosomal DNA transcription by preventing PHF6-mediated enrichment of repressive histone mark H4K20me3. J. Biol. Chem. 297 (4), 101195. 10.1016/j.jbc.2021.101195 34520760 PMC8511956

[B29] Hui B. Pan S. Che S. Sun Y. Yan Y. Guo J. (2021). Silencing UHRF1 enhances radiosensitivity of esophageal squamous cell carcinoma by inhibiting the PI3K/Akt/mTOR signaling pathway. Cancer Manag. Res. 13, 4841–4852. 10.2147/cmar.S311192 34188537 PMC8232844

[B30] Ilango S. Paital B. Jayachandran P. Padma P. R. Nirmaladevi R. (2020). Epigenetic alterations in cancer. Front. Biosci. Landmark Ed. 25 (6), 1058–1109. 10.2741/4847 32114424

[B31] Jacquet K. Binda O. (2021). ING proteins: tumour suppressors or oncoproteins. Cancers (Basel) 13 (9), 2110. 10.3390/cancers13092110 33925563 PMC8123807

[B32] Jain K. Fraser C. S. Marunde M. R. Parker M. M. Sagum C. Burg J. M. (2020). Characterization of the plant homeodomain (PHD) reader family for their histone tail interactions. Epigenetics Chromatin 13 (1), 3. 10.1186/s13072-020-0328-z 31980037 PMC6979384

[B33] Jeong D. W. Park J. W. Kim K. S. Kim J. Huh J. Seo J. (2023). Palmitoylation-driven PHF2 ubiquitination remodels lipid metabolism through the SREBP1c axis in hepatocellular carcinoma. Nat. Commun. 14 (1), 6370. 10.1038/s41467-023-42170-0 37828054 PMC10570296

[B34] Jian Y. Wang M. Zhang Y. Ou R. Zhu Z. Ou Y. (2018). Jade family PHD finger 3 (JADE3) increases cancer stem cell-like properties and tumorigenicity in colon cancer. Cancer Lett. 428, 1–11. 10.1016/j.canlet.2018.04.012 29660380

[B35] Karmakar S. Rauth S. Nallasamy P. Perumal N. Nimmakayala R. K. Leon F. (2020). RNA polymerase II-associated factor 1 regulates stem cell features of pancreatic cancer cells, independently of the PAF1 complex, via interactions with PHF5A and DDX3. Gastroenterology 159 (5), 1898–1915. 10.1053/j.gastro.2020.07.053 32781084 PMC7680365

[B36] Li P. Sun J. Ruan Y. Song L. (2021). High PHD Finger Protein 19 (PHF19) expression predicts poor prognosis in colorectal cancer: a retrospective study. PeerJ 9, e11551. 10.7717/peerj.11551 34141488 PMC8176917

[B37] Li Y. Li H. (2012). Many keys to push: diversifying the 'readership' of plant homeodomain fingers. Acta Biochim. Biophys. Sin. (Shanghai) 44 (1), 28–39. 10.1093/abbs/gmr117 22194011

[B38] Liu Q. Borcherding N. C. Shao P. Maina P. K. Zhang W. Qi H. H. (2020). Contribution of synergism between PHF8 and HER2 signalling to breast cancer development and drug resistance. EBioMedicine 51, 102612. 10.1016/j.ebiom.2019.102612 31923801 PMC7000350

[B39] Liu Q. Pang J. Wang L. A. Huang Z. Xu J. Yang X. (2021a). Histone demethylase PHF8 drives neuroendocrine prostate cancer progression by epigenetically upregulating FOXA2. J. Pathol. 253 (1), 106–118. 10.1002/path.5557 33009820 PMC7756255

[B40] Liu R. Gao J. Yang Y. Qiu R. Zheng Y. Huang W. (2018). PHD finger protein 1 (PHF1) is a novel reader for histone H4R3 symmetric dimethylation and coordinates with PRMT5-WDR77/CRL4B complex to promote tumorigenesis. Nucleic Acids Res. 46 (13), 6608–6626. 10.1093/nar/gky461 29846670 PMC6061854

[B41] Liu X. Zhang Z. Kan S. Lv Z. Zhou S. Liu X. (2021b). PHF20 inhibition promotes apoptosis and cisplatin chemosensitivity via the OCT4-p-STAT3-MCL1 signaling pathway in hypopharyngeal squamous cell carcinoma. Int. J. Oncol. 59 (1), 38. 10.3892/ijo.2021.5218 33982773 PMC8121096

[B42] Liu Y. Chen T. Guo M. Li Y. Zhang Q. Tan G. (2021c). FOXA2-Interacting FOXP2 prevents epithelial-mesenchymal transition of breast cancer cells by stimulating E-cadherin and PHF2 transcription. Front. Oncol. 11, 605025. 10.3389/fonc.2021.605025 33718155 PMC7947682

[B43] Maina P. K. Shao P. Liu Q. Fazli L. Tyler S. Nasir M. (2016). c-MYC drives histone demethylase PHF8 during neuroendocrine differentiation and in castration-resistant prostate cancer. Oncotarget 7 (46), 75585–75602. 10.18632/oncotarget.12310 27689328 PMC5342763

[B44] Mantovani F. Collavin L. Del Sal G. (2019). Mutant p53 as a guardian of the cancer cell. Cell Death Differ. 26 (2), 199–212. 10.1038/s41418-018-0246-9 30538286 PMC6329812

[B45] Mao S. Li Y. Lu Z. Che Y. Huang J. Lei Y. (2019). PHD finger protein 5A promoted lung adenocarcinoma progression via alternative splicing. Cancer Med. 8 (5), 2429–2441. 10.1002/cam4.2115 30932358 PMC6536992

[B46] Melekhova A. Baniahmad A. (2021). ING tumour suppressors and ING splice variants as coregulators of the androgen receptor signalling in prostate cancer. Cells 10 (10), 2599. 10.3390/cells10102599 34685579 PMC8533759

[B47] Melekhova A. Leeder M. Pungsrinont T. Schmäche T. Kallenbach J. Ehsani M. (2021). A novel splice variant of the inhibitor of growth 3 lacks the plant homeodomain and regulates epithelial-mesenchymal transition in prostate cancer cells. Biomolecules 11 (8), 1152. 10.3390/biom11081152 34439818 PMC8392754

[B48] Miller T. C. Rutherford T. J. Birchall K. Chugh J. Fiedler M. Bienz M. (2014). Competitive binding of a benzimidazole to the histone-binding pocket of the Pygo PHD finger. ACS Chem. Biol. 9 (12), 2864–2874. 10.1021/cb500585s 25323450 PMC4330097

[B49] Mohammad H. P. Barbash O. Creasy C. L. (2019). Targeting epigenetic modifications in cancer therapy: erasing the roadmap to cancer. Nat. Med. 25 (3), 403–418. 10.1038/s41591-019-0376-8 30842676

[B50] Moubarak R. S. de Pablos-Aragoneses A. Ortiz-Barahona V. Gong Y. Gowen M. Dolgalev I. (2022). The histone demethylase PHF8 regulates TGFβ signaling and promotes melanoma metastasis. Sci. Adv. 8 (7), eabi7127. 10.1126/sciadv.abi7127 35179962 PMC8856617

[B51] Murphy C. Gornés Pons G. Keogh A. Ryan L. McCarra L. Jose C. M. (2023). An analysis of JADE2 in non-small cell lung cancer (NSCLC). Biomedicines 11 (9), 2576. 10.3390/biomedicines11092576 37761019 PMC10526426

[B52] Oh S. Boo K. Kim J. Baek S. A. Jeon Y. You J. (2020). The chromatin-binding protein PHF6 functions as an E3 ubiquitin ligase of H2BK120 via H2BK12Ac recognition for activation of trophectodermal genes. Nucleic Acids Res. 48 (16), 9037–9052. 10.1093/nar/gkaa626 32735658 PMC7498345

[B53] Okuno Y. Ohtake F. Igarashi K. Kanno J. Matsumoto T. Takada I. (2013). Epigenetic regulation of adipogenesis by PHF2 histone demethylase. Diabetes 62 (5), 1426–1434. 10.2337/db12-0628 23274892 PMC3636657

[B54] Pan G. Zhang K. Geng S. Lan C. Hu X. Li C. (2022). PHF14 knockdown causes apoptosis by inducing DNA damage and impairing the activity of the damage response complex in colorectal cancer. Cancer Lett. 531, 109–123. 10.1016/j.canlet.2022.01.002 35074497

[B55] Pappa S. Padilla N. Iacobucci S. Vicioso M. Álvarez de la Campa E. Navarro C. (2019). PHF2 histone demethylase prevents DNA damage and genome instability by controlling cell cycle progression of neural progenitors. Proc. Natl. Acad. Sci. U. S. A. 116 (39), 19464–19473. 10.1073/pnas.1903188116 31488723 PMC6765295

[B56] Sahai E. Astsaturov I. Cukierman E. DeNardo D. G. Egeblad M. Evans R. M. (2020). A framework for advancing our understanding of cancer-associated fibroblasts. Nat. Rev. Cancer 20 (3), 174–186. 10.1038/s41568-019-0238-1 31980749 PMC7046529

[B57] Schindler U. Beckmann H. Cashmore A. R. (1993). HAT3.1, a novel Arabidopsis homeodomain protein containing a conserved cysteine-rich region. Plant J. 4 (1), 137–150. 10.1046/j.1365-313x.1993.04010137.x 8106082

[B58] Strikoudis A. Lazaris C. Trimarchi T. Galvao Neto A. L. Yang Y. Ntziachristos P. (2016). Regulation of transcriptional elongation in pluripotency and cell differentiation by the PHD-finger protein Phf5a. Nat. Cell Biol. 18 (11), 1127–1138. 10.1038/ncb3424 27749823 PMC5083132

[B59] Taheri M. Hussen B. M. Najafi S. Abak A. Ghafouri-Fard S. Samsami M. (2022). Molecular mechanisms of inhibitor of growth (ING) family members in health and malignancy. Cancer Cell Int. 22 (1), 272. 10.1186/s12935-022-02693-w 36056353 PMC9438315

[B60] Toh T. B. Lim J. J. Chow E. K. (2017). Epigenetics in cancer stem cells. Mol. Cancer 16 (1), 29. 10.1186/s12943-017-0596-9 28148257 PMC5286794

[B61] Wagner E. K. Nath N. Flemming R. Feltenberger J. B. Denu J. M. (2012). Identification and characterization of small molecule inhibitors of a plant homeodomain finger. Biochemistry 51 (41), 8293–8306. 10.1021/bi3009278 22994852 PMC3567257

[B62] Wang Y. Hu P. Wang F. Xi S. Wu S. Sun L. (2023). UHRF1 inhibition epigenetically reprograms cancer stem cells to suppress the tumorigenic phenotype of hepatocellular carcinoma. Cell Death Dis. 14 (6), 381. 10.1038/s41419-023-05895-w 37380646 PMC10307895

[B63] Wang Z. Yang X. Liu C. Li X. Zhang B. Wang B. (2019). Acetylation of PHF5A modulates stress responses and colorectal carcinogenesis through alternative splicing-mediated upregulation of KDM3A. Mol. Cell 74 (6), 1250–1263. 10.1016/j.molcel.2019.04.009 31054974

[B64] Weissman A. M. (2001). Themes and variations on ubiquitylation. Nat. Rev. Mol. Cell Biol. 2 (3), 169–178. 10.1038/35056563 11265246

[B65] Wu S. Luo C. Li F. Hameed N. U. F. Jin Q. Zhang J. (2019). Silencing expression of PHF14 in glioblastoma promotes apoptosis, mitigates proliferation and invasiveness via Wnt signal pathway. Cancer Cell Int. 19, 314. 10.1186/s12935-019-1040-6 31798343 PMC6882144

[B66] Wu X. Chen C. Luo B. Yan D. Yan H. Chen F. (2020). Nuclear ING3 expression is correlated with a good prognosis of breast cancer. Front. Oncol. 10, 589009. 10.3389/fonc.2020.589009 33469513 PMC7813678

[B67] Xiaoyun S. Yuyuan Z. Jie X. Yingjie N. Qing X. Yuezhen D. (2021). PHF19 activates hedgehog signaling and promotes tumorigenesis in hepatocellular carcinoma. Exp. Cell Res. 406 (1), 112690. 10.1016/j.yexcr.2021.112690 34129846

[B68] Yang L. Shi P. Zhao G. Xu J. Peng W. Zhang J. (2020). Targeting cancer stem cell pathways for cancer therapy. Signal Transduct. Target Ther. 5 (1), 8. 10.1038/s41392-020-0110-5 32296030 PMC7005297

[B69] Yang Y. Li M. Zhou X. Wang W. Shao Y. Yao J. (2022). PHF5A contributes to the maintenance of the cancer stem-like phenotype in non-small cell lung cancer by regulating histone deacetylase 8. Ann. Clin. Lab. Sci. 52 (3), 439–451. 35777798

[B70] Yu Q. Yin L. Jian Y. Li P. Zeng W. Zhou J. (2019). Downregulation of PHF6 inhibits cell proliferation and migration in hepatocellular carcinoma. Cancer Biother Radiopharm. 34 (4), 245–251. 10.1089/cbr.2018.2671 30888215

[B71] Yuan S. Wang X. Hou S. Guo T. Lan Y. Yang S. (2022). PHF6 and JAK3 mutations cooperate to drive T-cell acute lymphoblastic leukemia progression. Leukemia 36 (2), 370–382. 10.1038/s41375-021-01392-1 34465864 PMC8807395

[B72] Zhao Y. He J. Li Y. Xu M. Peng X. Mao J. (2020). PHF14 promotes cell proliferation and migration through the AKT and ERK1/2 pathways in gastric cancer cells. Biomed. Res. Int. 2020, 6507510. 10.1155/2020/6507510 32596345 PMC7305535

[B73] Zheng Y. Z. Xue M. Z. Shen H. J. Li X. G. Ma D. Gong Y. (2018). PHF5A epigenetically inhibits apoptosis to promote breast cancer progression. Cancer Res. 78 (12), 3190–3206. 10.1158/0008-5472.Can-17-3514 29700004

[B74] Zhu Z. Y. Tang N. Wang M. F. Zhou J. C. Wang J. L. Ren H. Z. (2021). Comprehensive pan-cancer genomic analysis reveals PHF19 as a carcinogenic indicator related to immune infiltration and prognosis of hepatocellular carcinoma. Front. Immunol. 12, 781087. 10.3389/fimmu.2021.781087 35069553 PMC8766761

